# Accuracy of Marginal and Internal Adaptation of Advanced Lithium Disilicate Crowns Using Different Margin Designs (In Vitro Study)

**DOI:** 10.1155/ijod/5550877

**Published:** 2026-03-03

**Authors:** Hossameldin Hassan Abdelrazek Mohamed, Amir Azer, Rewaa G. AboElHassan

**Affiliations:** ^1^ Department of Conservative Dentistry, Fixed Prosthodontics, Faculty of Dentistry, Alexandria University, Alexandria, Egypt, alexu.edu.eg

**Keywords:** CAD/CAM, ceramics, crown, crown preparations, marginal adaptation

## Abstract

**Background:**

Advanced lithium disilicate glass ceramics offer excellent esthetics, but conservative crown preparations are not always the preferred choice. Various margin designs are available; however, more conservative vertical preparations (vertipreps) have less scientific evidence supporting their esthetic outcomes compared to shoulder or chamfer designs.

**Aim of the Study:**

To assess the marginal adaptation and internal fit of crowns fabricated from advanced lithium disilicate (CEREC Tessera) using computer‐aided design and manufacturing (CAD/CAM) with three distinct margin designs.

**Materials and Methods:**

Three mandibular molar typodont teeth were prepared with three finish line designs: chamfer 0.5 mm (C), rounded shoulder 1 mm (RS), and vertiprep 0.2 mm (V) using a modified dental surveyor for standardization. Silicone replicas were used to create epoxy resin dies. Twenty‐four crowns were produced on the replicated resin dies, with eight crowns for each finish line design (*n* = 8). Preparations were scanned using an intraoral scanner (Carestream Dental LLC, 3625 Cumberland Blvd., Ste. 700, Atlanta, GA 30339), and advanced lithium disilicate (Tessera) crowns were milled via CAD/CAM. Crowns were cemented onto their respective dies, and marginal adaptation was measured using a stereomicroscope; then, crowns were sectioned using a microtome to evaluate internal fit.

**Results:**

One‐way ANOVA showed a significant difference in the marginal adaptation among groups (*p*  < 0.001). Group RS had the largest gap (125.45 ± 12.11 µm), followed by Group C (107.31 ± 9.25 µm) and Group V (101.79 ± 9.01 µm). Internal fit also differed significantly (*p*  < 0.001), with Group V having the smallest gap (70.09 ± 8.45 µm), followed by Group RS (87.85 ± 6.82 µm) and Group C (94.45 ± 9.21 µm).

**Conclusion:**

The vertiprep showed the least marginal adaptation and best internal fit compared to shoulder and chamfer designs, with no significant difference in marginal gap between vertiprep and chamfer.

## 1. Background

The accuracy of CAD/CAM restorations is strongly influenced by both the preparation design and the milling process. Well‐prepared teeth with clear margins allow the system to produce crowns with better adaptation, while poor preparations increase marginal discrepancies. Marginal adaptation depends on the precision of the finish line reproduction, since large gaps can lead to cement breakdown or secondary caries. Internal fit is also preparation‐dependent, as smooth internal surfaces facilitate accurate seating of the restoration. Overall, tooth preparation quality and CAD/CAM accuracy work together to determine crown longevity and clinical success [1, 2].

Marginal adaptation and internal fit of restorations are two important clinical factors in assessing the quality and durability of CAD/CAM ceramic restorations. The misfits between the restoration and the prepared tooth, including marginal adaptation and internal fit affecting the restoration seating, lead to microleakage, recurrent caries, tooth hypersensitivity, followed by periodontal disease and eventual failure of restorations [3]. The clinically acceptable range of marginal adaptation, less than 174 µm, was reported in systematic reviews [4, 5], and the values of the internal fit were between 200 and 300 µm [6].

In general, there are two types of preparation: horizontal preparation, which uses finish lines, and nonhorizontal preparation, referred to as featheredge or vertical preparation (vertiprep) [7]. The shoulder and chamfer are commonly used as horizontal finish lines in dental preparations due to their widespread application. This approach helps to minimize overhangs and overcontouring, which are notable drawbacks of vertiprep. However, vertiprep designs have been restricted due to challenges in accurately locating the tapered, thin margins and the increased risk of chipping fractures in the restorations [8, 9].

The design of crown preparation plays a key role in selecting the restorative material, as it affects retention, support, esthetics, and the necessary thickness for strength. Less invasive vertipreps are generally suited for ceramic restorations, whereas more extensive horizontal designs are often required for materials like zirconia, which offer superior mechanical strength. Because materials vary in fracture resistance and stiffness, the preparation must be planned to harmonize material properties with the biological needs of the tooth [10]. New material, advanced lithium disilicate CAD/CAM blocks (CEREC Tessera), was introduced by Dentsply Sirona in 2021. Advanced lithium disilicate glass ceramic contains a two‐part crystal composition (lithium disilicate + virgilite) embedded in a glassy zirconia matrix; the new ceramic composition made it possible for the ceramic to fire quickly for 4.5–12 min at 760°C. Virgilite is a new crystal type that is activated by the matrix firing process. The flexural strength of advanced lithium disilicate is over 700 MPa. It exhibited better results in marginal adaptation, internal fit, and fracture resistance compared to traditional lithium disilicate crowns [11]. It would be a promising material regarding the marginal integrity and fracture resistance of the crown [12, 13]. The present study aims to evaluate the marginal adaptation and the internal fit of advanced lithium disilicate crowns prepared with different forms of finish lines: rounded shoulder (RS), chamfer (C), and vertiprep (V). The null hypothesis was that no significant difference would be found between the three different finish line designs.

## 2. Material and Methods

### 2.1. Study Design and Sample Size

This study was an in vitro, parallel‐controlled trial aimed at evaluating the marginal gap and internal fit among three different groups. The study was conducted in the laboratory of the Conservative Dentistry Department at the Faculty of Dentistry, Alexandria University, Egypt.

The sample size was determined using the 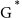
Power software (Version 3.1.9.2, Department of Biomedical Informatics and Medical Statistics, Medical Research Institute, University of Alexandria, Egypt), with assumptions of 80% statistical power and a 5% alpha level. Based on mean comparisons from a previous study, the required sample size was calculated as 8 specimens per group. The total sample size required = number of groups × number per group = 3 × 8 = 24 [14].

### 2.2. Specimen Preparation

Three mandibular left second molar typodont teeth (Kilgore Nissin 200 brand, USA) were used to prepare three finish line designs to receive advanced lithium disilicate (CEREC Tessera: Dentsply Sirona, USA) crowns.

**Figure 1:**
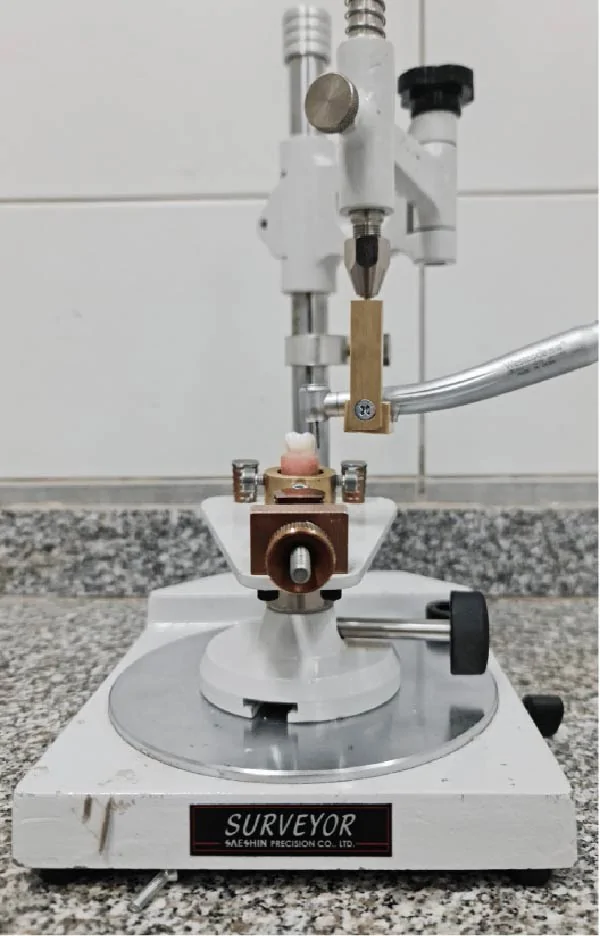
Modified dental surveyor.

Three mandibular teeth were inserted into acrylic blocks and prepared with the following criteria:

Around 1.5–2 mm occlusal reduction for all groups, and the axial reduction was 0.5 mm for group C, 1 mm for group RS, and 0.2 mm for group V, with a total convergence angle of 6° using a modified dental surveyor for standardization and to ensure a consistent degree of taper [1, 15, 16] (Figures 1 and 2).

Figure 2(a) Chamfer, (b) shoulder, and (c) vertiprep.

**Figure:**
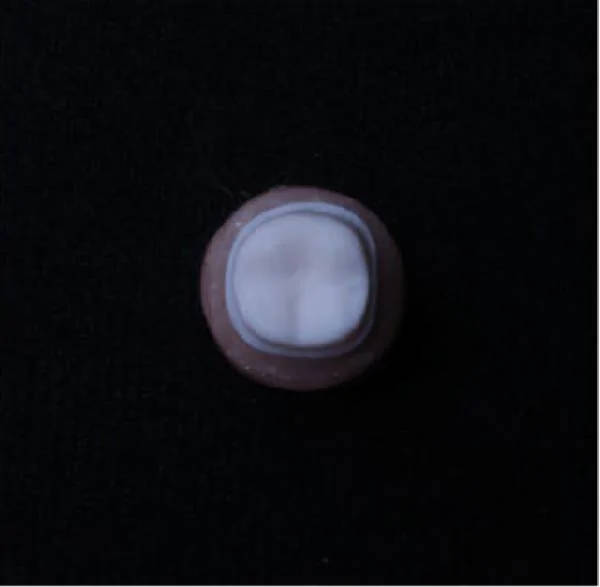
(a)

**Figure:**
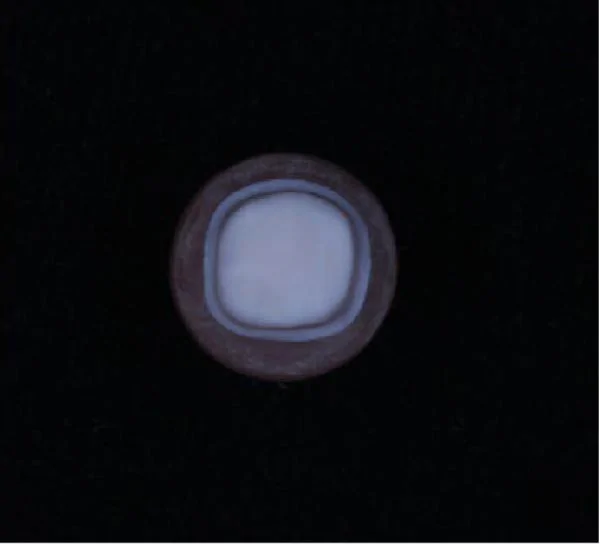
(b)

**Figure:**
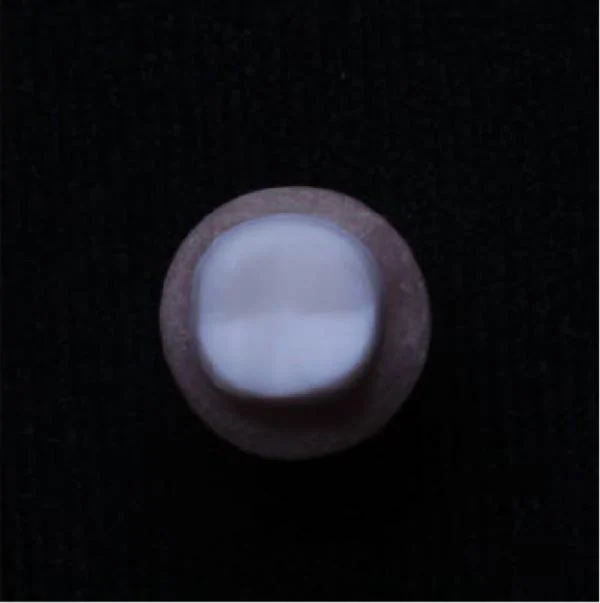
(c)

Positive replicas of the prepared teeth were fabricated for each preparation by using a silicone duplicating material. Subsequently, epoxy resin material (RenCast Epoxy Casting Resin, CH‐4057 Basel, Switzerland) was poured eight times for each group, with a total number of 24 models across all groups [17]. These epoxy dies were then categorized into three groups according to their respective finish line designs.

Twenty‐four epoxy resin dies were scanned using an intraoral scanner (Carestream Dental LLC, 3625 Cumberland Blvd., Ste. 700, Atlanta, GA 30339), and the scan data were then imported into CAD software (Exocad 3.0, Galway GmbH, Darmstadt, Germany) to design the crown, with the cement space set at 50 µm for the axial and occlusal surfaces for better fit accuracy and adequate fracture strength [18–20]. After designing, the STL file was transmitted to a computer‐controlled five‐axis milling unit (ED5X EMAR, C2, Industrial Complex, 10th of Ramadan City, Asharqia, Egypt) for the milling procedure, and each crown was milled to its corresponding scanned model (Figure 3).

Figure 3Scanning of specimens with different designs. (a) Chamfer, (b) shoulder, and (c) vertiprep.

**Figure:**
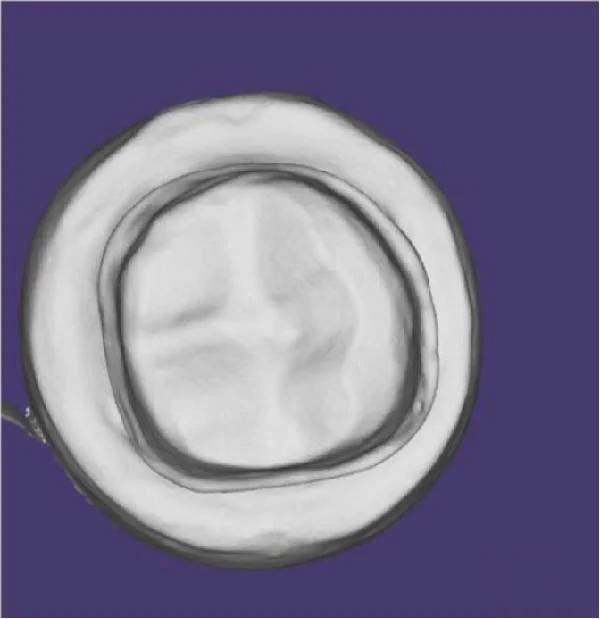
(a)

**Figure:**
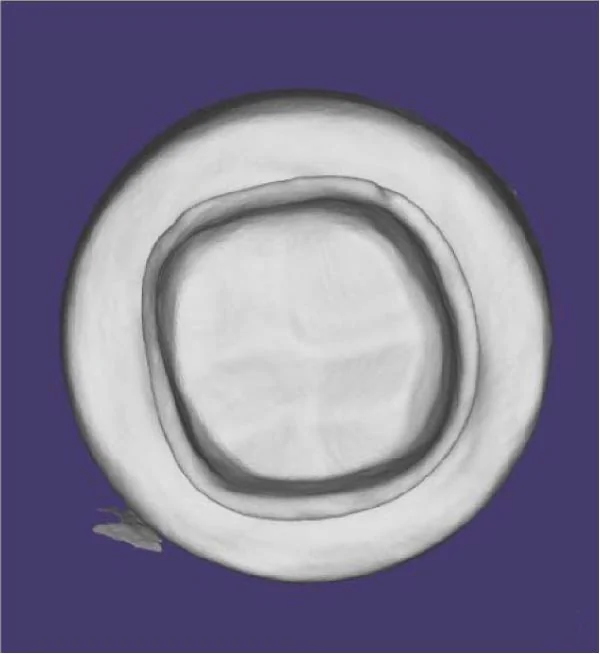
(b)

**Figure:**
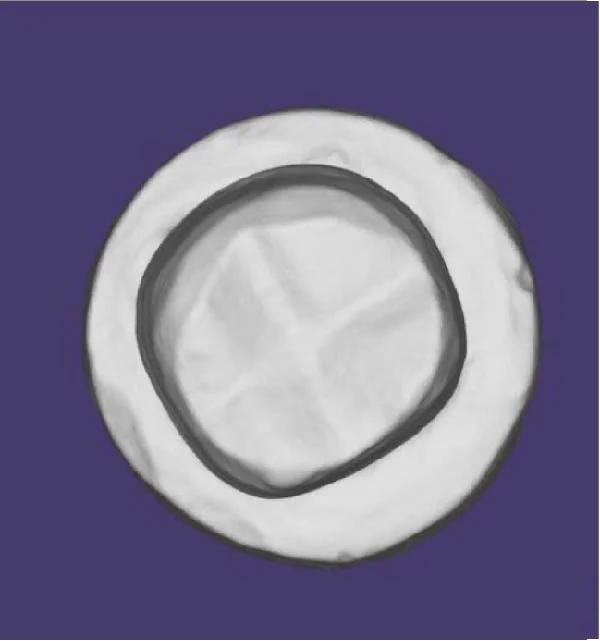
(c)

**Figure 4:**
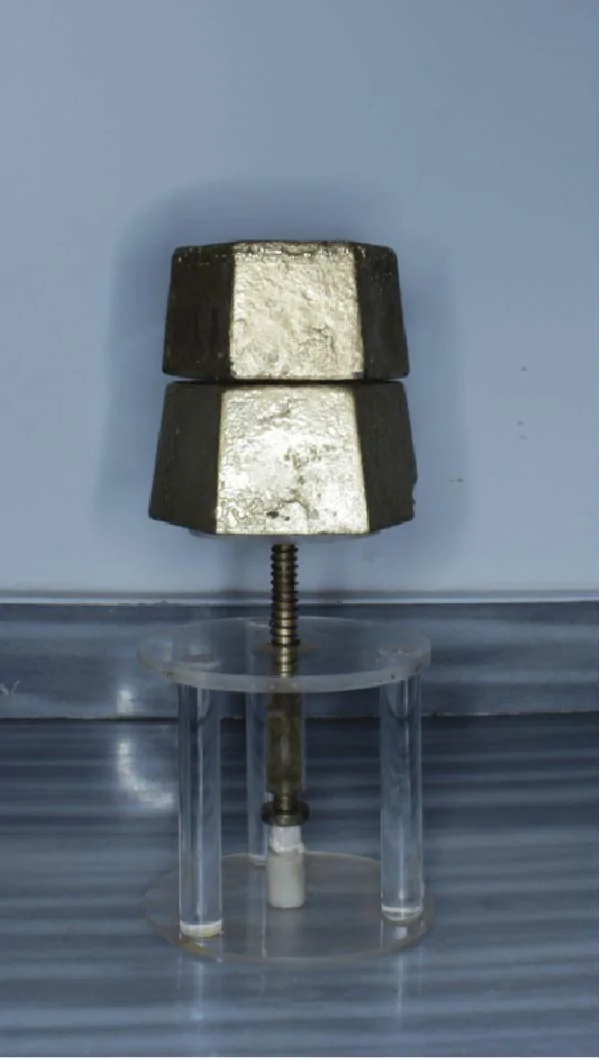
Loading 5 kg on the specimen.

**Figure 5:**
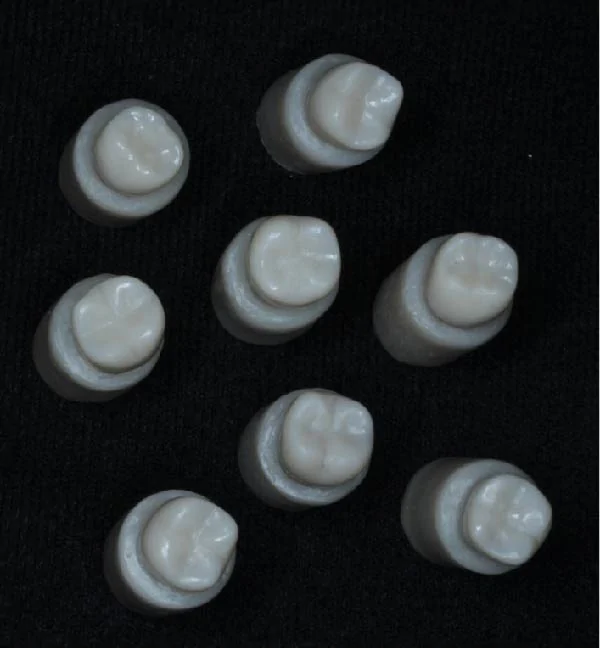
Grouping samples after cementation of Tessera crowns on their corresponding dies.

Each crown was then cemented to its corresponding die after etching the intaglio surfaces of the crowns using hydrofluoric acid gel 9.5% (BISCO’s PORCELAIN ETCHANT 9.5% Hydrofluoric Acid Gel), following the manufacturer’s recommendations. Silane (BISCO‐Schaumburg, USA) was applied to the bonding surfaces and left for 60 s, then air‐dried for 5 s. Crowns were bonded using dual‐cure adhesive resin cement (Supercem Self Etch, Self‐Adhesive Resin Cement Universal, DENTKIST, KOREA). They were positioned on the corresponding dies using finger pressure, then subjected to an axial static load of 5 kg, followed by an initial light‐curing for 1–2 s to remove excess cement with a scalpel blade, followed by a final curing exposure of 40 s on each surface of the crown, and the chemical setting time was 5 min [1] (Figures 4 and 5).

To ensure full polymerization, all specimens were kept at 37°C and 100% relative humidity for 24 h then thermocycled [21] 2500 times to simulate 3 months of clinical use by a custom‐made device, Dental Biomaterial Department, Faculty of Dentistry, Alexanderia University. Each water bath had a dwell time of 25 s, along with a 10 s delay, and temperatures ranged from a minimum of 5°C to a maximum of 55°C [21, 22].

### 2.3. Evaluation of Marginal Gap and Internal Adaptation

Before sectioning of crowns, the vertical marginal gap was evaluated for each group; each specimen was evaluated separately by using a stereomicroscope (Olympus SV, Japan) connected to a digital camera under a magnification of 40× [1, 23, 24].

**Figure 6:**
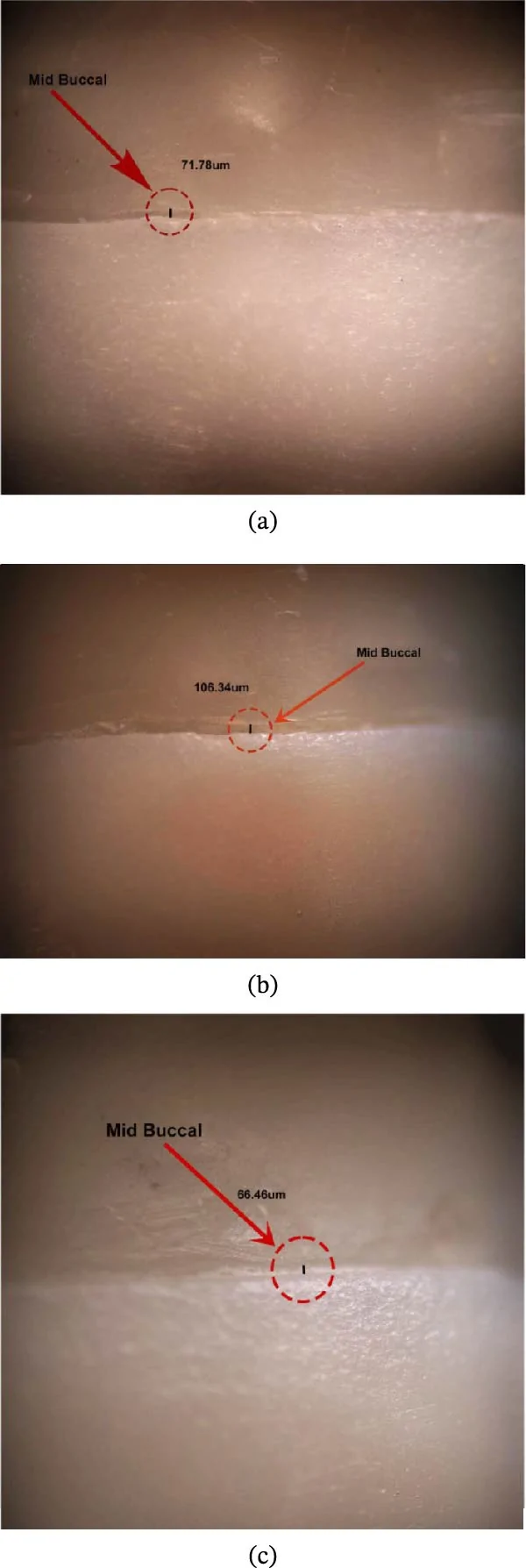
Marginal gap in midbuccal captured by stereomicroscope at 40× magnification. (a) Chamfer design, (b) rounded shoulder, and (c) vertical preparation design.

**Figure 7:**
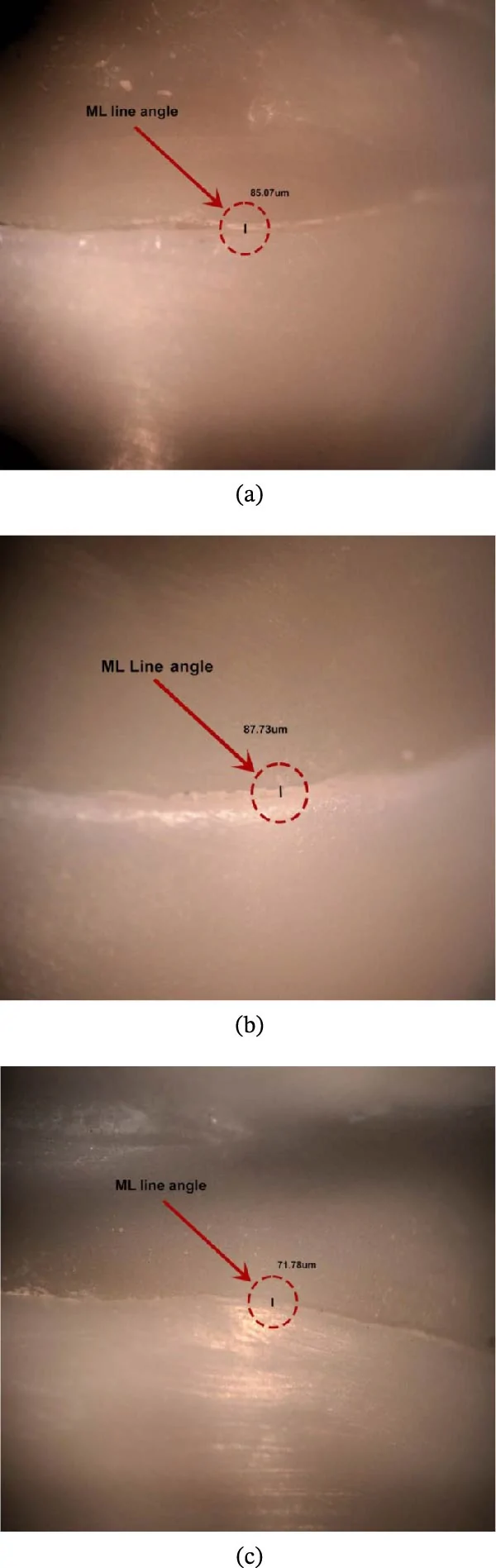
Marginal gap in mesiolingual line angle captured by stereomicroscope at 40× magnification. (a) Chamfer design, (b) rounded shoulder, and (c) vertical preparation design.

Eight points were selected (midbuccal, midlingual, middistal, midmesial, mesiolingual, mesiobuccal, distolingual, and distobuccal) to be measured by a stereomicroscope [23–27]. All measurements were carried out by a single blind examiner (Figures 6 and 7).

**Figure 8:**
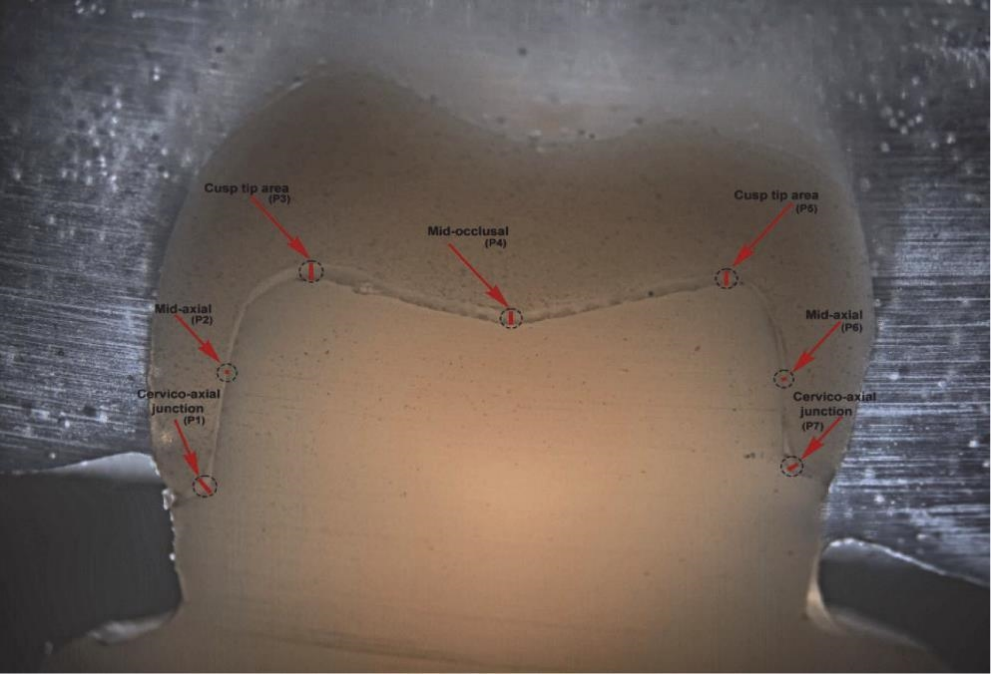
Internal gaps presentation in specimen after sectioning.

**Figure 9:**
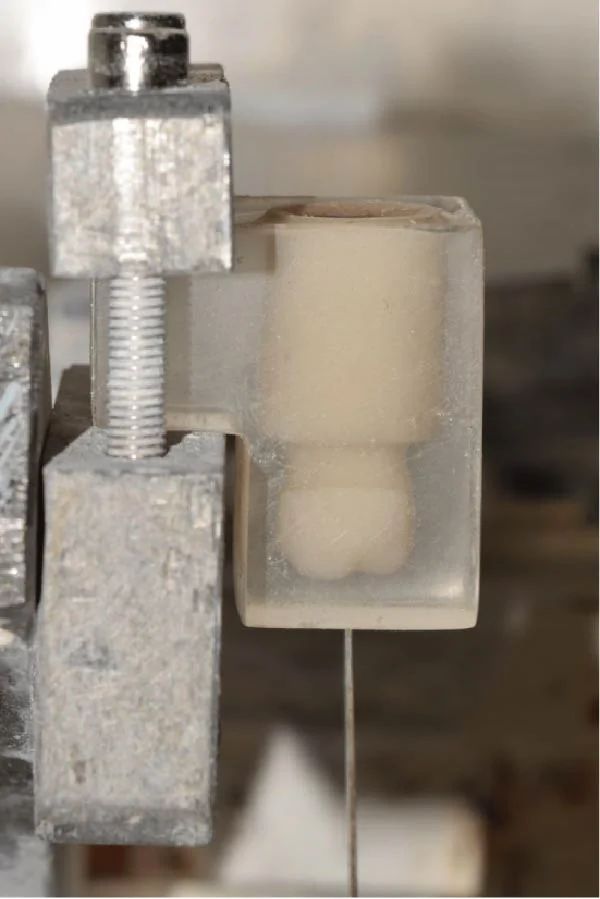
Specimen in acrylic block attached to microtome for sectioning in buccolingual direction.

**Figure 10:**
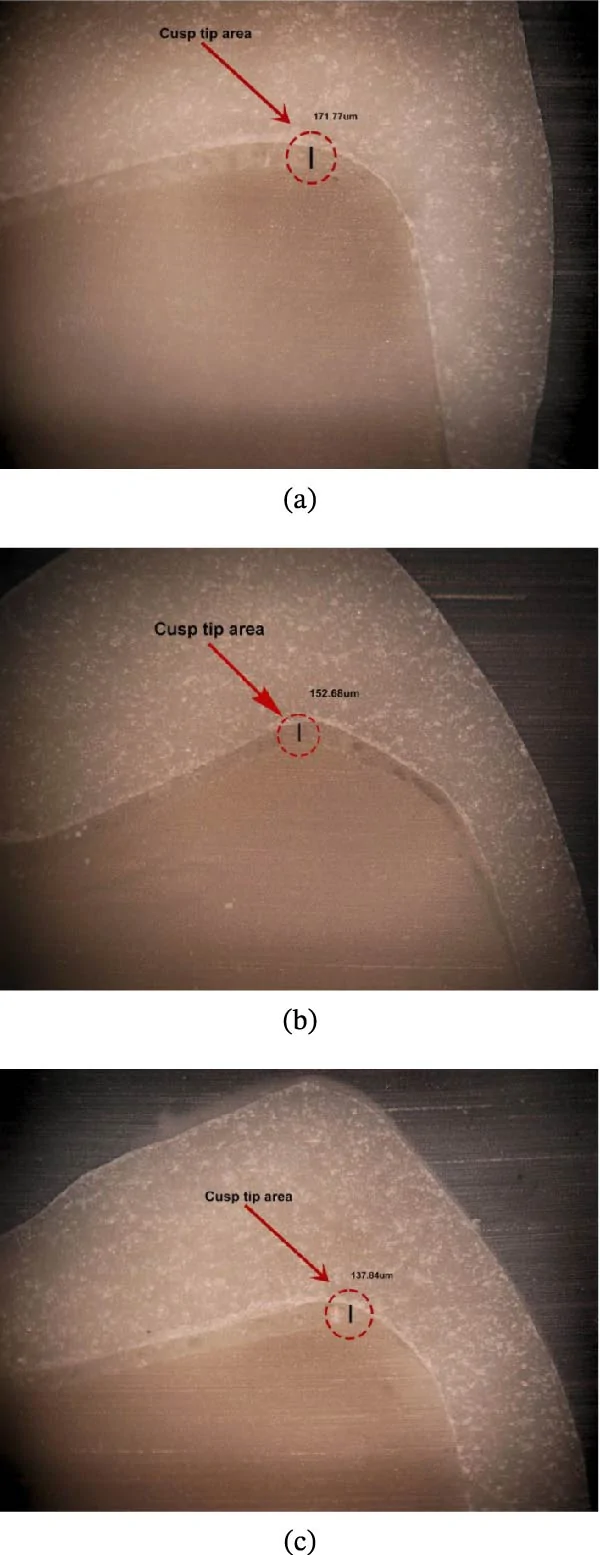
Internal gap of cusp tip area captured by stereomicroscope at 60× magnification. (a) Chamfer design, (b) rounded shoulder, and (c) vertical preparation design.

**Figure 11:**
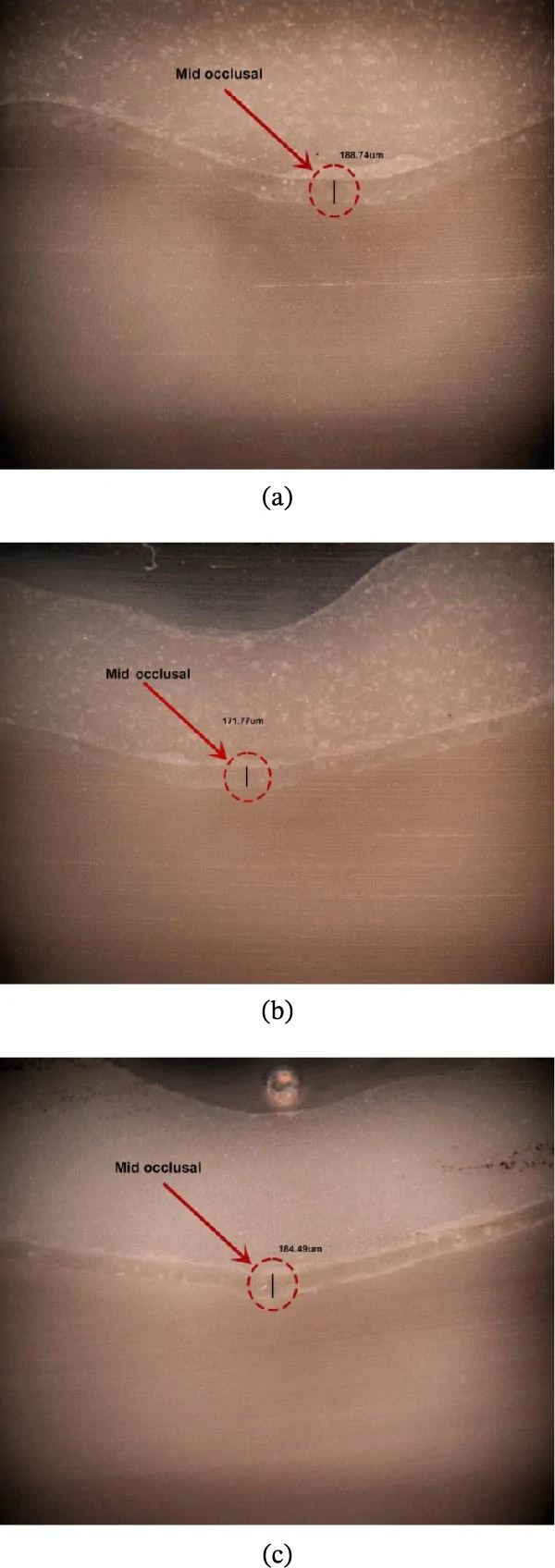
Internal gap of midocclusal point captured by stereomicroscope at 60× magnification. (a) Chamfer design, (b) rounded shoulder, and (c) vertical preparation design.

The specimens were placed in transparent epoxy blocks and sectioned at the midplane buccolingually by a microtome (Micracut 150, Metkon Metallography Bursa, Turkey), then cleaned by micro brush to remove any potential debris that could affect the measurements and evaluated for the internal fit at the following seven points of each group under magnification of 60× using a stereomicroscope [25, 28, 29] (Figures 8–11).

P1: The junction of the cervical and axial wall on the buccal aspect.
P2: The midaxial point on the buccal aspect.
P3: Cusp tip area.
P4: The midocclusal point.
P5: Cusp tip area.
P6: The midaxial point on the lingual aspect.
P7: The junction of the cervical and axial wall on the lingual aspect.

### 2.4. Statistical Analysis

Data were analyzed using IBM SPSS, version 23, Armonk, NY, USA. The normality of variables was checked using the Shapiro–Wilk test and Q–Q plots. Normal distribution was confirmed for both marginal gap and internal adaptation; thus, values were mainly presented using mean and standard deviation (SD) in addition to median, minimum, and maximum values. Comparison between groups was done using one‐way ANOVA followed by Tukey’s post hoc test with Bonferroni correction to adjust for Type I error. All tests were two‐tailed, and the significance level was set at α = 0.05.

## 3. Results

**Figure 12:**
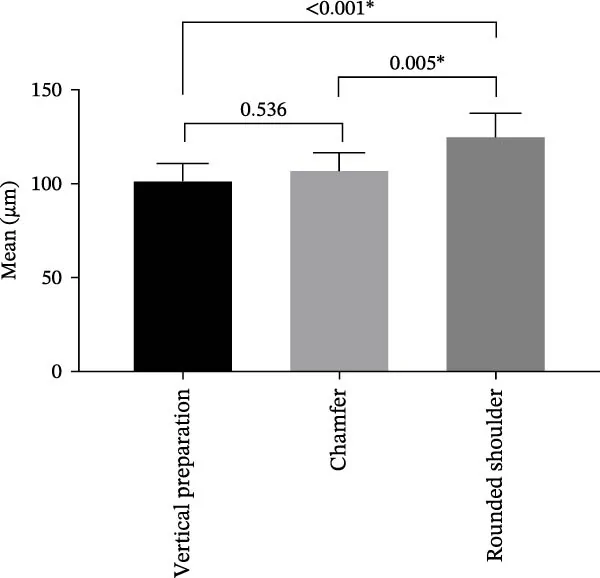
Comparison of marginal gap (µm) among the study groups.

For the marginal adaptation (µm) among the groups. The group RS preparation technique resulted in the largest mean marginal adaptation, followed by group C and group V (Table 1) (Figure 12).

**Table 1:** Comparison of marginal adaptation (µm) among the study groups.

Statistical parameters	Vertiprep (*n* = 8)	Chamfer (*n* = 8)	Rounded shoulder (*n* = 8)
Mean ± SD	101.79 ± 9.01	107.31 ± 9.25	125.45 ± 12.11
Median	99.95	109.44	130.12
Min – max	91.62 – 122.61	94.33 – 119.71	108.64 – 136.60
*F*‐test (*p*‐value)	11.73 (<0.001 ^∗^)		

^∗^Statistically significant difference at *p*‐value < 0.05, *F*‐test: One‐way ANOVA test.

Post hoc pairwise comparisons revealed that there was no significant difference between the V and C groups. In contrast, the V and RS groups differed significantly. Similarly, the C and RS groups also showed a significant difference (Table 2) (Figure 12).

**Table 2:** Pairwise comparisons between study groups regarding marginal adaptation.

Groups	Compared to	Mean diff	95% CI	*p*‐Value^a^
Vertiprep	Chamfer	−5.52	−18.40, 7.36	0.536
	Rounded shoulder	−23.66	−36.54, −10.77	<0.001 ^∗^
Chamfer	Rounded shoulder	5.52	−7.36, 18.40	0.005 ^∗^

Abbreviation: CI, confidence interval.

^a^ *p* Value: Tukey’s post hoc test.

^∗^Statistically significant difference at *p* value < 0.05.

**Figure 13:**
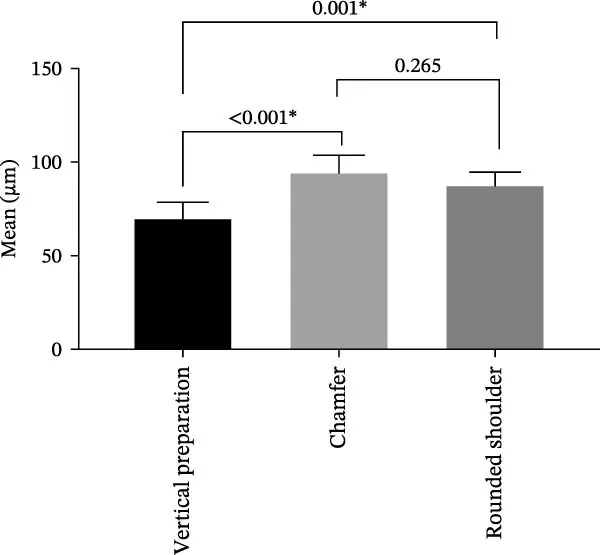
Comparison of internal adaptation (µm) among the study groups.

For the internal fit (µm) across the three preparation techniques. The V group had the smallest mean internal adaptation, followed by the RS group and the C group, which had the largest mean (Figure 13) (Table 3).

**Table 3:** Comparison of internal fit (µm) among the study groups.

Statistical parameters	Vertiprep (*n* = 8)	Chamfer (*n* = 8)	Rounded shoulder (*n* = 8)
Mean ± SD	70.09 ± 8.45	94.45 ± 9.21	87.85 ± 6.82
Median	72.06	95.02	85.69
Min – max	57.91 – 82.50	74.74 – 106.18	82.64 – 104.34
*F*‐test (*p*‐value)	18.81 (<0.001 ^∗^)		

^∗^Statistically significant difference at *p*‐value < 0.05. *F*‐test: One‐way ANOVA test.

Post hoc pairwise comparisons indicated that the V and C groups differed significantly. The V and RS groups also showed a significant difference. However, the difference between the C and RS groups was not statistically significant (Table 4) (Figure 13).

**Table 4:** Pairwise comparisons between study groups regarding internal fit.

Groups	Compared to	Mean diff	95% CI	*p*‐Value^a^
Vertiprep	Chamfer	−24.37	−34.73, −14.01	<0.001 ^∗^
	Rounded shoulder	−17.76	−28.12, −7.40	0.001 ^∗^
Chamfer	Rounded shoulder	24.37	14.01, 34.72	0.265

Abbreviation: CI, confidence interval.

^a^ *p* Value: Tukey’s post hoc test.

^∗^Statistically significant difference at *p* value < 0.05.

## 4. Discussion

The marginal adaptation and the internal fit of advanced lithium disilicate crowns (CEREC Tessera) were evaluated among three different marginal designs. The null hypothesis was rejected, as significant differences were found between Group V and Group RS in marginal adaptation and between Group V and Group C in internal fit. However, no significant difference was observed between Group V and Group C in marginal adaptation, nor between Group C and Group RS in internal fit. These results highlight the influence of preparation design on the marginal and internal adaptation of advanced lithium disilicate crowns.

Compared to horizontal preparations, the vertiprep design provided a clearly superior coronal seal and reduced the gap between the teeth and the crown, improving the fit and reducing bacterial penetration. This effect is attributed to the vertical configuration of the preparation. As the marginal angle decreases from 90° shoulders to 45° or 30° chamfers and finally to 0° vertical margins, the gap becomes progressively smaller. In theory, vertical margins can result in an almost perfect, gap‐free fit [30–32].

CAD/CAM material development follows two paths: enhancing zirconia’s translucency without losing strength and improving glass ceramics’ strength while retaining esthetics. These advances may converge in materials like advanced lithium disilicate (e.g., CEREC Tessera), which combines strength and esthetics, can be etched for durable bonding, and supports more conservative procedures [33, 34]. In the context of s, such material characteristics are highly influential. The ability to achieve a strong adhesive interface contributes to precise marginal adaptation, reducing microgaps at the finish line, while the superior machinability of glass ceramics enhances the accuracy of CAD/CAM milling, thereby improving the internal fit of restorations [35]. Consequently, the balance of esthetics, strength, and bonding not only supports functional and esthetic outcomes but also directly impacts the quality of adaptation in vertipreps, reinforcing the rationale for the chosen restorative material in this study.

To ensure standardization, all types of crown preparations were carried out using a modified dental surveyor. For all samples, a dual‐cured universal adhesive resin cement was utilized for luting all crown restorations onto their respective epoxy resin dies. These dies were selected for their dimensional stability and a modulus of elasticity (11.8 GPa), which closely approximates that of dentin (18 GPa), and showing provided crown restorations with acceptable marginal fit [36, 37].

Thermocycling was used to simulate 3 months of clinical service, in accordance with International Organization for Standardization guidelines. Thermal aging produced only a minimal increase in marginal discrepancies, which remained within clinically acceptable limits [19, 38].

The marginal adaptation of all crowns was evaluated using a stereomicroscope set at a fixed 40× magnification for measuring the marginal gap and 60× magnification for measuring the internal fit. This technique provides a cost‐effective and nondestructive method for assessment, allowing measurements to be performed directly without the need for intermediate materials such as impression material or luting cement. However, this technique also presents certain limitations, including challenges in accurately identifying measurement points at the margin, difficulties in reproducing these points consistently, and problems in clearly distinguishing the tooth structure from the restoration, especially in shaded areas [1, 23–25].

For internal fit evaluation, the cross‐sectional method (CSM) was a widely utilized technique for evaluating crown fit and marginal adaptation. It provided precise measurements directly from the prosthesis after cementation, effectively simulating clinical conditions. It’s exhibited for its accuracy and high‐resolution imaging; this conventional approach ensured reliable assessment, and besides, it is methodologically efficient [39, 40].

So that each sample was sectioned in buccallingual directions along the long axis of the crown using a microtome [28]. A study compared the CSM with the silicon replica technique (SRT), which did not find statistically significant differences between the values obtained with CSM and SRT [40].

In the present study, there was a significant difference in the marginal adaptation among the three different groups. The rounded shoulder preparation technique showed the largest mean marginal adaptation (125.45 ± 12.11 µm), followed by the chamfer with a mean marginal gap (107.31 ± 9.25 µm) and vertiprep, which exhibited the least mean marginal adaptation (101.79 ± 9.01 µm); however, there was no significant difference between the chamfer and vertiprep designs.

The reference range of marginal adaptation reported in our literature was between 91.62 and 136.60 µm; these values fall within the clinically acceptable range, which is less than 174 µm as, reported in systematic reviews [4, 5].

In previous studies, the number of measurement points per crown used has varied considerably; the present results were compatible with Comlekoglu et al. [41], who measured 16 points per crown and found that the vertiprep had the highest marginal adaptation in comparison with the round shoulder finish line in zirconia crowns. According to these findings, this was because the more the restoration margin finishes with an acute angle, the smaller the marginal adaptation, as previously explained by Schillinburg et al. [42].

The current results were aligned with Salama et al. [19], who measured marginal adaptation in zirconia crowns with vertical design as opposed to those with rounded shoulder design; they concluded that specimens with a rounded shoulder finish line exhibited significantly higher marginal adaptation values compared to those with featheredge finish lines.

Similarly, it was consistent with El‐Eneen et al. [43], who reported lower marginal adaptation values for the vertiprep design than chamfer finish lines in zirconia‐reinforced lithium silicate crowns, but it was statistically not significant, and Eldamaty et al. [44], who discovered no statistically significant differences in the marginal accuracy of monolithic zirconia crowns with chamfer and vertical margins.

In contrast with the present findings, Nasir and Kadhim [45] evaluated the influence of the chamfer and vertiprep techniques on marginal adaptation in zirconia crowns. The study demonstrated that there was a significant difference between the vertiprep group, which demonstrated the smallest marginal adaptation values, and the chamfer group, which showed the largest marginal adaptation values. Different results were obtained because the study was based on extracted natural teeth from orthodontic patients.

Furthermore, Rizonaki et al. [46] reported that the rounded shoulder finish line had the least marginal gap between the chamfer and vertiprep design in lithium disilicate crowns (e.max CAD), and the notable differences may be revealed due to using a different methodology, which is microcomputed tomography, which may create artificial defects caused by ray reflections. However, the micro‐CT obscures marginal spaces in narrow gaps [40, 47].

There were significant differences in the internal fit among the three different groups. Vertiprep showed the smallest mean internal fit (70.09 ± 8.45 µm), followed by rounded shoulder (87.85 ± 6.82 µm), and chamfer, which exhibited the largest mean (94.45 ± 9.21 µm). However, there was no significant difference between shoulder and chamfer designs.

The reference range of internal fit reported in our literature review was between 57.91 and 106.18 µm. These values fall within the clinically acceptable range, which is between 200 and 300 µm [6].

The current findings were supported by Rizonaki et al. [46], who reported that the vertiprep design had the best internal fit compared with shoulder and chamfer finish line design preparation in lithium disilicate crowns.

It was consistent with ElgGandy et al. [48], who found that there was no significant difference in the internal fit values between rounded shoulder and chamfer designs in lithium disilicate crowns.

Also aligned with Baig et al. [49], whose results reported that there was no statistically significant difference in internal fit values between shoulder and chamfer finish lines in lithium disilicate crowns.

In contrast to current findings, Al‐Baadani et al. [50] found that the internal fit of full contour zirconia crowns with a deep chamfer finish line design was better than full contour zirconia crowns with a shoulder finish line design, and this may be due to the fact that the thickness of the deep chamfer design was 1 mm.

Furthermore, Rizonaki et al. [46] showed that the rounded shoulder group exhibited the highest internal fit values compared to the chamfer and vertiprep groups; this discrepancy may result because the cement space was set at 80 µm.

In the present study, advanced lithium disilicate strengthened with virgilite (CEREC Tessera) was used for the milling of all crowns. More than 700 MPa flexural strength, with up to a 32% increase in strength compared to conventional lithium disilicate, was claimed by the manufacturer, as the interwoven dual crystal composition between lithium disilicate and virgilite crystals acts like a reinforcement bar (rebar effect), and this might have contributed to the high‐margin adaptation of the vertiprep design after both milling and thermomechanical aging in comparison to the chamfer margin, as the risk of chipping was reduced [13, 51].

This study had several limitations. Notably, evaluating marginal adaptation in vivo is challenging, as luting agents and bonding mechanisms can influence marginal adaptation and internal fit, especially with temperature fluctuations [22, 52]. It is recommended that advanced technologies, such as the triple‐scan protocol or micro‐CT scanning, be employed in future studies [46].

## 5. Conclusion

Vertiprep design revealed the least marginal adaptation and internal fit compared to rounded shoulder designs for advanced lithium disilicate crowns (CEREC Tessera). However, there was no significant difference between the vertiprep and the chamfer design in marginal adaptation. This suggests that VertiPrep may be a more conservative and biologically compatible approach, as it preserves more tooth structure while maintaining excellent adaptation.

**Nomenclature**
°C: Celsius or centigrade
µm: Micrometer
CAD/CAM: Computer‐aided design and computer‐aided manufacturing
IBM SPSS: Software for advanced statistical analysis
kg: Kilogram
mm: Millimeter
MO: Marginal opening
MPa: Megapascal
no.: Number
P: Probability
SD: Standard deviation.

## Author Contributions

Hossameldin Hassan Abdelrazek Mohamed contributed to the study design, the data acquisition, analysis, and interpretation, and wrote the manuscript draft. Rewaa G. AboElHassan contributed to the study design and the interpretation of the results and revised the manuscript. Amir Azer contributed to the study design, revised the manuscript, and supervised the work.

## Funding

This study was not funded by any agency in the public, commercial, or nonprofit sectors.

## Disclosure

All authors have read and approved the final manuscript.

## Ethics Statement

This study was approved by the Scientific Research Ethics Committee at the Faculty of Dentistry, Alexandria University (International Number: IORG0008839, Ethics Committee Number: 0488‐8/2023). All methods were performed following the Declaration of Helsinki and the ethical guidelines adopted by the Research Ethics Committee of the Faculty of Dentistry, Alexandria University. No human participants were involved in the study.

## Consent

The authors have nothing to report.

## Conflicts of Interest

The authors declare no conflicts of interest.

## Data Availability

The datasets generated and analyzed during the current study are available from the corresponding author upon reasonable request.
